# Pembrolizumab combined with surgical treatment for spontaneously ruptured undifferentiated pleomorphic sarcoma of the liver: a case report and literature review

**DOI:** 10.3389/fimmu.2025.1691575

**Published:** 2025-11-10

**Authors:** Tao He, Sun Ke, Jieyu Zou

**Affiliations:** 1 Department of Hepatobiliary Surgery, Chengdu Second People’s Hospital, Chengdu, Sichuan, China; 2 Department of Oncology, Chengdu Second People’s Hospital, Chengdu, Sichuan, China

**Keywords:** pembrolizumab, surgical treatment (debridement or necrosectomy), liver sarcoma, immunotherapy, prognosis

## Abstract

Undifferentiated pleomorphic sarcoma of the liver (UPSL) is a rare pathological type characterized by an undefined mechanism, low incidence, high metastatic rate, aggressive behavior, and an inferior prognosis; no standardized treatment protocols or guidelines currently exist. This article reports the case of an 83-year-old male with UPSL confirmed through surgical resection and pathological biopsy. Postoperatively, he received eight cycles of pembrolizumab, which resulted in a favorable clinical efficacy. With advances in medical technology, the integration of surgery and immunotherapy is expected to play an essential role in treating this rare disease and monitoring its prognosis.

## Introduction

Cancer arises primarily from somatic or germline oncogenic alterations in oncogenes and tumor suppressor genes. Diverse tumor types exist, and most exhibit multiple oncogenic alterations with considerable heterogeneity both within and between individual tumors (1). As a genomic disease, cancer is characterized by genomic instability and the progressive accumulation of numerous point mutations and structural alterations (2). These genomic changes can generate tumor antigens that are recognized by the immune system as foreign, thereby initiating cellular immune responses (3). Consequently, the immune system performs a vital function in immune surveillance. Throughout cancer evolution, the accumulation of point mutations and structural variants not only drives malignant transformation but also enhances the immunogenicity of cancer cells (4). Mutated gene products can be identified by the host immune system as non-self antigens, leading to immune-mediated clearance (5). Immunotherapy seeks to augment the body’s inherent defenses to eliminate malignant cells. This transformative strategy has profoundly changed oncology practice. Major categories of immunotherapy include several modalities that have shown clinical potential: oncolytic virus therapy (6), cancer vaccines (7), cytokine therapy such as interferon (8), adoptive cell transfer including chimeric antigen receptor (CAR)-T-cell therapies (9), and immune checkpoint inhibitors (ICIs)including pembrolizumab therapies (10). ICIs work by blocking common inhibitory signaling pathways to reactivate anti-tumor immunity and facilitate immune-mediated destruction of malignant cells (11). The most frequently targeted molecules are cytotoxic T lymphocyte-associated antigen 4 (CTLA-4), programmed cell death protein 1 (PD-1), and programmed death ligand-1 (PD-L1) (12–14).

Undifferentiated pleomorphic sarcoma (UPS), previously known as malignant fibrous histiocytoma (MFH), was first reported in 1964 by O’Brien and Stout (15). UPS arises most frequently in the limbs and only rarely involves the liver, which predominantly affects middle-aged and elderly adults and demonstrates high rates of both postoperative recurrence and distant metastasis (16). Pathological examination and immunohistochemical staining are essential for a definitive diagnosis for UPS. Owing to the rarity of the disease in liver, no standardized treatment protocol currently exists. This report presents a case of UPS of the liver (UPSL) managed with surgical resection combined with immunotherapy, which yielded a favorable therapeutic response. We aim to offer effective perspectives on the management of this rare tumor through this approach.

## Case description

**Figure 1:**
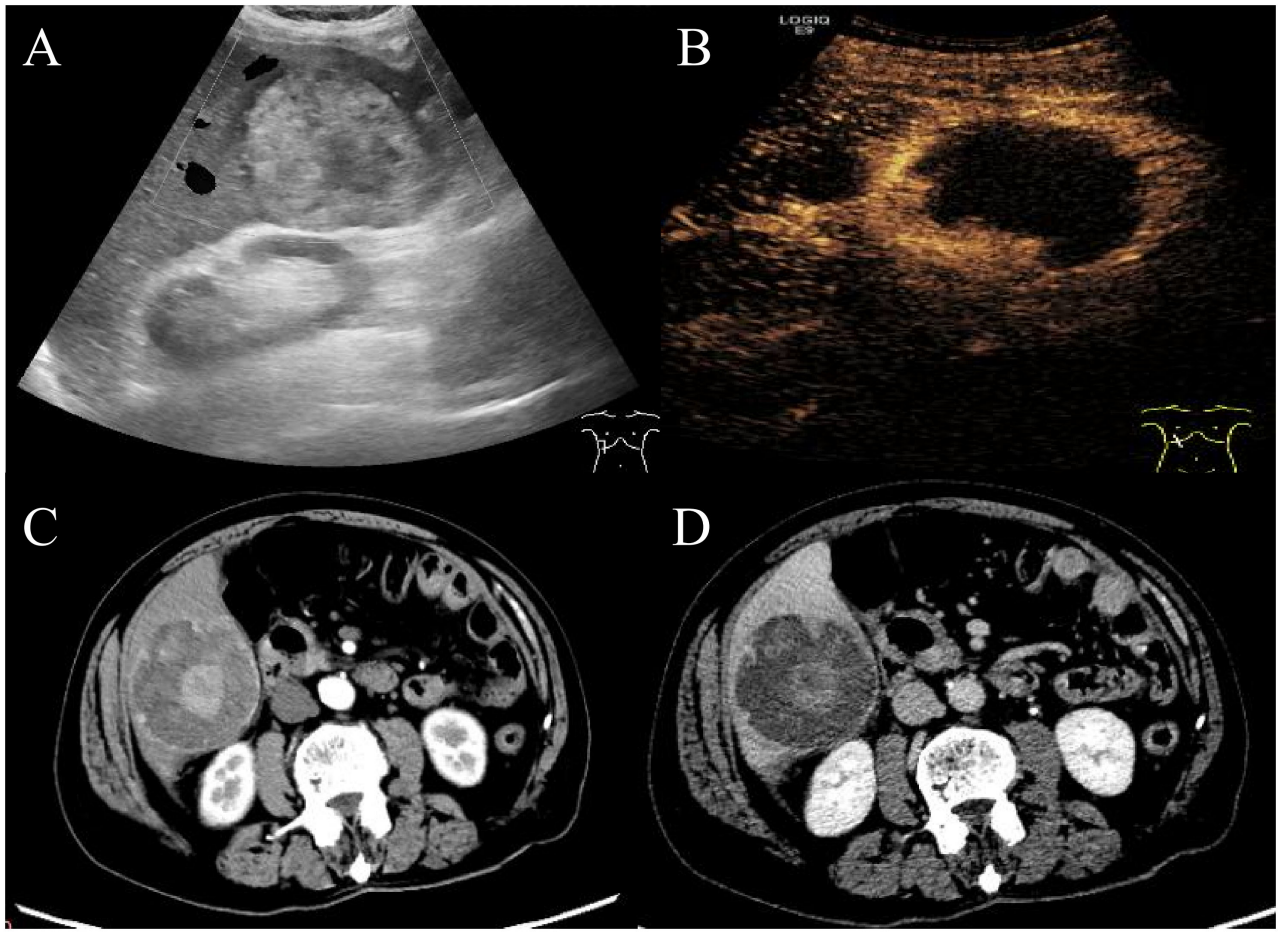
Abdominal ultrasound and enhanced CT imaging of the manifestations of liver lesions. Abdominal ultrasound and contrast-enhanced ultrasound showed a well-defined hyperechoic lesion in the right posterior hepatic lobe **(A, B)**. Contrast-enhanced CT revealed a hyperechoic tumor in hepatic segment VI with peripheral hyperdensity and central hypodensity with heterogeneous hyperdensity during the portal phase **(C, D)**.

**Figure 2:**
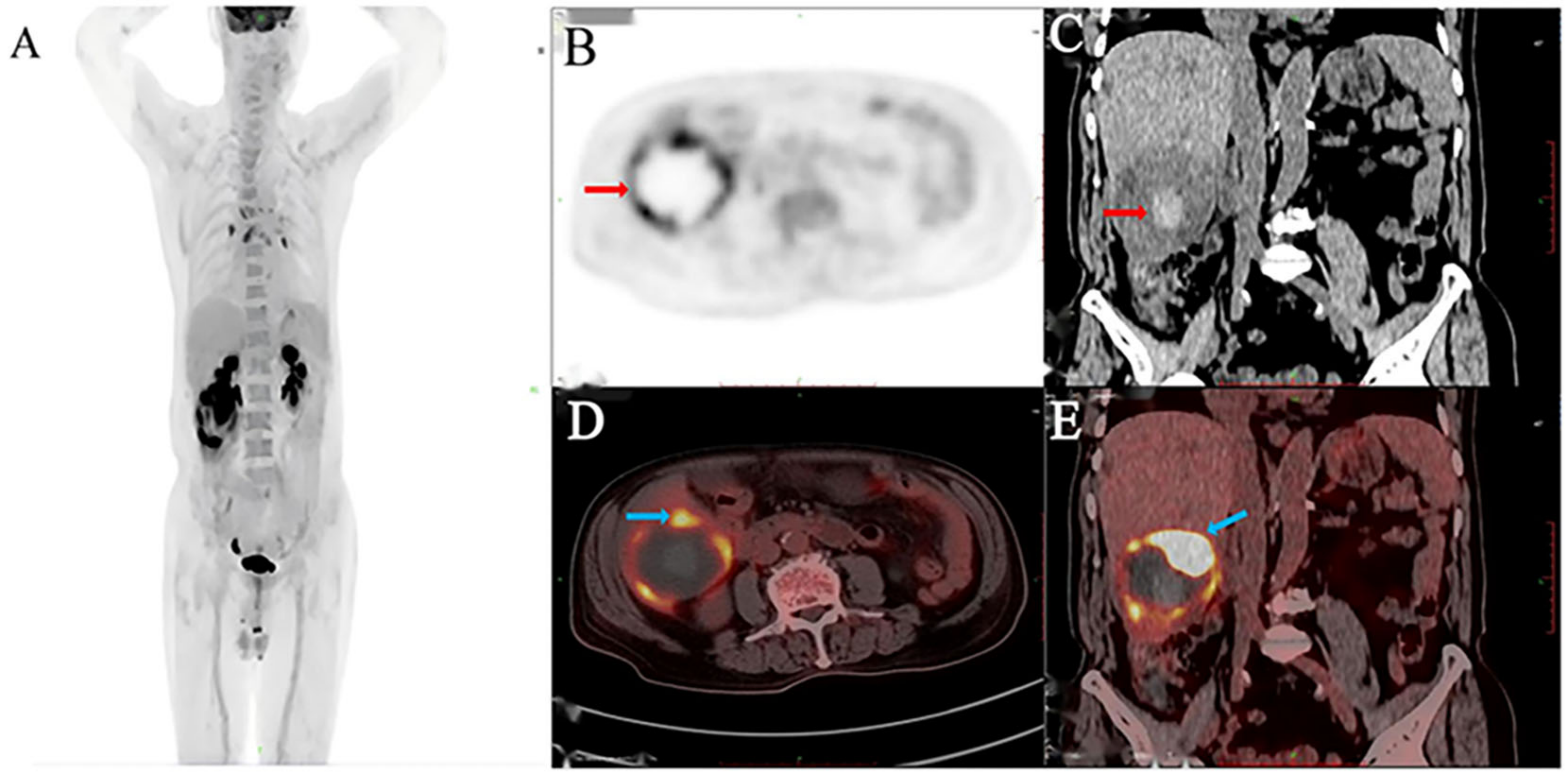
18F-FDG PET/CT imaging identified a hepatic malignancy. The MIP image **(A)**, PET **(B)**, CT **(C)**, and axial **(D)** and coronal **(E)** fused images revealed a hypermetabolic right hepatic tumor with an SUVmax of 21.4.

An 83-year-old man presented with acute upper abdominal pain and distension lasting one day, with no history of trauma. He had a 20-year history of chronic hepatitis B virus(HBV) infection managed with daily entecavir (0.5 mg). He previously underwent surgical treatment with stent implantation for coronary atherosclerotic heart disease. Physical examination demonstrated right upper quadrant tenderness without peritoneal signs, Murphy’s sign, venous collaterals, or ascites. Laboratory findings revealed mild anemia (hemoglobin 94 g/L), with reduced red blood cell count (3.44 × 10¹²/L), hematocrit (31%), and mean corpuscular hemoglobin concentration (302 g/L). The ultrasensitive C-reactive protein level was markedly elevated at 100.16 mg/L. Liver function tests showed modestly increased gamma-glutamyl transferase and alkaline phosphatase (146 U/L each), while albumin measured 32.5 g/L with normal aminotransferase levels. Coagulation studies indicated a prothrombin time of 14.1 seconds (INR 1.32). Serology confirmed hepatitis B surface antigen positivity with undetectable viral DNA (<20 IU/mL). The tumor marker AFP was elevated (853 ng/ml), while other markers such as CA99, CA125, CEA and PIVKA-II were unremarkable. Abdominal ultrasound identified a well-circumscribed, mildly hyperechoic tumor in hepatic segment VI displaying heterogeneous echotexture (**Figure 1A**). Contrast-enhanced ultrasound revealed a well-defined hyperechoic lesion in the right posterior hepatic lobe, which strongly suggest hepatic malignancy (**Figure 1B**). Contrast-enhanced CT suggested a 6.7 × 6.8 × 6.5 cm mass in the same segment, showing peripheral hyperdensity and central hypodensity with heterogeneous enhancement during the portal phase (**Figures 1C, D**). The maximum intensity projection (MIP) image detected multiple pulmonary nodules and a lesion at the gastric fundus. Bilateral adrenal glands and several bone marrow sites exhibited mildly increased 18F-FDG uptake, with maximum SUVs of 1.1, 3.2, 2.4, and 3.2. These findings suggested benign rather than metastatic involvement (**Figure 2A**). 18F-fluorodeoxyglucose-positron emission tomography (18F-FDG-PET)/CT showed a heterogeneous mass in the right hepatic lobe (**Figure 2B***)* that displaced the hepatic capsule outward and displayed intense metabolic activity (**Figure 2C***)*, with markedly elevated 18F-FDG uptake and a maximum standardized uptake value (SUV) of 21.4 (**Figures 2D, E**).

**Figure 3:**
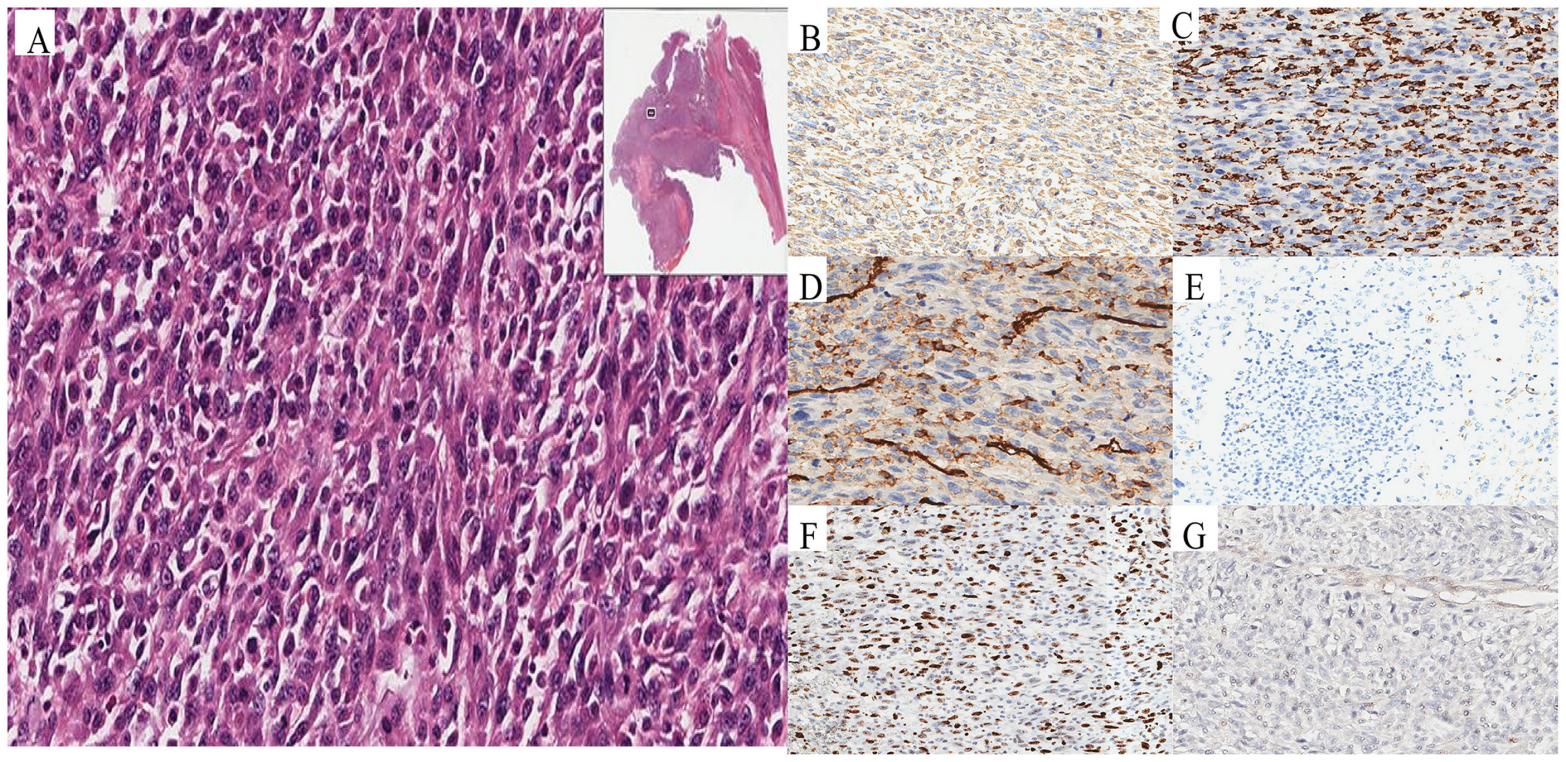
Pathological and immunohistochemical characteristics of UPSL. Histopathology revealed malignant characteristics, including tumor necrosis, spindle and epithelioid cell morphology, and elevated mitotic activity **(A)**. Immunohistochemical staining showed diffuse strong vimentin expression **(B)**, CD68 **(C)**, CD31 **(D)** and SMA positivity **(E)**, Ki-67 proliferation **(F)**, and weak PD-L1 reactivity **(G)**.

**Figure 4:**
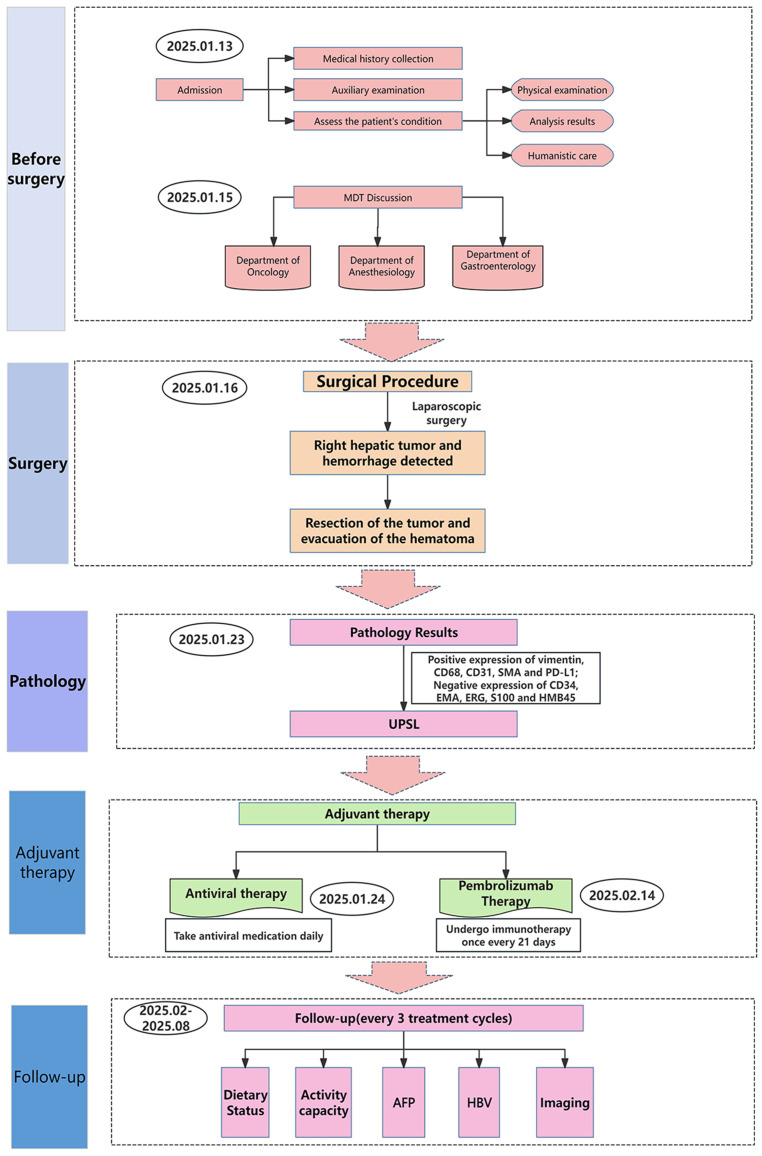
The flowchart of diagnosis and treatment for this patient.

Following multidisciplinary team evaluation, the liver lesion was diagnosed as malignant, with hepatocellular carcinoma (HCC) considered the probable diagnosis. The patient subsequently underwent right hepatic tumor resection. Intraoperative exploration revealed a ruptured hemorrhagic tumor firmly adherent to the right abdominal wall and transverse colon, with an organized hematoma in the right subphrenic space. Consequently, the surgical procedure included both tumor resection and evacuation of the rupture-induced hematoma. Notably, histopathological analysis confirmed malignant features, including tumor cell necrosis, spindle and epithelioid morphology, and increased mitotic activity (**Figure 3A**). Immunohistochemistry(IHC) results (**Table 1**) demonstrated diffuse strong vimentin expression (**Figure 3B**), weak cytoplasmic CD68 (**Figure 3C**), focal CD31 (**Figure 3D**) and SMA positivity (**Figure 3E**), ki-67 proliferation index of 70% (**Figure 3F**), and weak programmed death ligand 1 (PD-L1) reactivity (**Figure 3G**). Remarkedly, IHC scoring indicates the percentage of PD-L1-positive tumor cells demonstrating significant membrane staining. If more than 1% of tumor cells exhibit membrane staining, the tumor is considered PD-L1-positive.Meanwhile, the tumor tested negative for CD34, EMA, ERG, S100, HMB45. Finally, these collective findings established a definitive diagnosis of spontaneously ruptured UPSL rather than HCC. Furthermore, the molecular test results indicated tumor suppressor p53(TP53) gene mutations and mutations in the promoter region of the telomerase reverse transcriptase (TERT) gene, but no mutations in the exons of the C-KIT and PDGFRA genes were observed. His clinical symptoms and laboratory markers improved significantly, enabling discharge while continuing entecavir therapy. Postoperatively, the patient refused to undergo chemotherapy for personal reasons and insisted on receiving immunotherapy instead. Following eight cycles of pembrolizumab (200 mg every three weeks) administered based on PD-L1 positive status, the patient has maintained clinical stability without detectable treatment-related adverse effects. **Figure 4** presents the patient’s diagnostic and treatment flowchart, and **Supplementary Figure 5** depicts the corresponding changes in AFP levels over the treatment period.

**Table 1:** The immunohistochemical results of the UPSL.

Immumohistochemical staining	Results
Vimentin	(+++)
CD68	(+)
SMA	(+)
CD31	(+)
ki-67	(+,70%)
CD34	(-)
CK7	(-)
EMA	(-)
S100	(-)
WT1	(-)
SOX-10	(-)
Desmin	(-)
Glypican-3	(-)
HMB45	(-)

(-), Negative; (+), Positive.

## Discussion

Since its initial documentation in 1985, fewer than 200 cases of UPSL have been reported (17, 18). Emerging evidence suggests that UPSL may originate from diverse cellular mutations, particularly those involving the PIK3/PTEN/AKT/mTOR and WNT/β-catenin pathways, which drive uncontrolled proliferation and transcriptional dysregulation (19, 20). Most UPSL cases are asymptomatic and are often discovered incidentally during physical examination, typically leading to further investigation when a liver mass is detected. When present, symptoms are nonspecific and may include right upper quadrant pain, weight loss, jaundice, fatigue, or anorexia (21, 22). Laboratory studies are generally unremarkable and contribute little to preoperative diagnosis. Although specific tumor markers are generally absent, one recent case reported a markedly elevated AFP level (1111.93 ng/mL) (18). The underlying cause of elevated AFP in UPSL remains unclear. Current evidence indicates a relationship with tumor dedifferentiation, during which tumor cells may aberrantly reactivate fetal gene expression programs and acquire the capacity to synthesize AFP (23). Serum AFP levels also correlate significantly with histological inflammatory activity and the degree of hepatic fibrosis, particularly in chronic hepatitis (24). In this case, increased AFP levels could stem from fibrosis, necrosis, and apoptosis triggered by the hepatitis B virus’s invasion of hepatocytes, despite a negative HBV DNA test, or they may result from tumor rupture. The decrease in AFP observed in this study is considered to be related to the following factors: 1. Surgical resection reduced tumor burden, and 2. Oral antiviral therapy slowed further viral replication in patients. In the paper, we also discuss the association between AFP and tumor-related factors.

The diagnosis of UPSL remains exceptionally challenging without pathological confirmation, as clinical manifestations, imaging findings, and tumor markers all lack standardization, leading to a high rate of misdiagnosis. Histopathological assessment is therefore essential for identifying liver tumors of uncertain origin. Macroscopically, UPS typically appears white to pale yellow and often contains central hemorrhage and necrosis (16). Histologically, the tumor is highly cellular and exhibits marked nuclear atypia, frequent mitotic figures, and areas of spindle cell morphology (16, 18). Remarkably, necrosis is commonly observed in high-grade lesions (16, 25). Although CD68 immunostaining is frequently positive in UPS, this marker is not diagnostically useful due to the absence of true histiocytic differentiation. Abundant tumor-infiltrating histiocytes may also contribute to false-positive CD68 interpretations (16). UPS is a diagnosis of exclusion that requires an extensive IHC panel to rule out other morphologically similar tumors. The differential diagnosis for mesenchymal tumors should exclude pleomorphic leiomyosarcoma, pleomorphic rhabdomyosarcoma, pleomorphic liposarcoma, and dedifferentiated liposarcoma. Pleomorphic leiomyosarcoma and rhabdomyosarcoma can be distinguished from UPS by their deeply eosinophilic cytoplasm, longitudinal myofibrils or cross-striations, and diffuse immunoreactivity for multiple myogenic markers (26). Pleomorphic liposarcoma often contains numerous pleomorphic giant cells, but its defining feature is the presence of multivacuolar pleomorphic lipoblasts, which may show positivity for CD34 and S100 (27). Dedifferentiated liposarcoma arises from well-differentiated components, is typically observed at the periphery of the lesion, and is characterized by mouse double minute 2(MDM2) gene amplification (28).

On the other hand, non-mesenchymal neoplasms that morphologically overlap with UPS are essentially sarcomatoid carcinoma, melanoma and gastrointestinal stromal tumors (GIST). Sarcomatoid carcinoma, an epithelial-derived malignancy that morphologically resembles sarcoma yet lacks its typical clinical and imaging features. It typically occurs in organs with epithelial tissue, such as the kidneys, the head and neck, the lungs, and the bladder (29). IHC showed strong and diffuse staining for keratin (particularly with multiple antibodies); expression of other epithelial markers, such as EMA or p63; and/or identification of intraepithelial dysplastic components (30). Melanoma is a malignant tumor originating from cutaneous melanocytes. Genomic alterations in components of the MAPK and PI3K pathways drive melanoma progression through these signaling cascades (31). Cytoplasmic pigment granules are frequently observed upon morphological examination. IHC serves as a key diagnostic tool, particularly through the detection of S100 and SOX10 proteins (31). Furthermore, UPS must be distinguished from GIST, which most commonly arise in the stomach or small intestine. Patients typically present with abdominal pain, gastrointestinal bleeding, or intestinal obstruction. Abdominal imaging reveals well-defined, highly vascular masses that often enhance heterogeneously in the arterial phase and may contain areas of hemorrhage or necrosis (32). IHC usually demonstrates expression of CD117 and DOG-1, while a subset of GIST also exhibits KIT gene amplification (33).

Surgical resection represents the primary approach for achieving curative treatment in non-metastatic UPS. The objective is to obtain complete excision with microscopically negative margins (R0 resection), as positive margins are strongly correlated with local recurrence (34). When anatomically feasible, a wide local excision with a margin of at least 1 cm is strongly recommended (35). Systemic therapy also plays a role in both resectable and metastatic UPS. Neoadjuvant or adjuvant chemotherapy has been shown to improve overall survival (OS), with doxorubicin plus ifosfamide (A + I) and gemcitabine plus docetaxel (G + D) among the most widely used regimens (16). Growing evidence supports the efficacy of doxorubicin-based adjuvant chemotherapy in high-risk UPS patients. The meta-analysis incorporated four additional trials utilizing intensified doxorubicin dosing combined with ifosfamide, revealing that adjuvant A + I reduced the absolute risk of death by 11% compared to no adjuvant treatment (36). Although the heterogeneity test was not significant (P = 0.46), the 18 included randomized controlled trials still displayed considerable variation. The chemotherapy regimens differed substantially, encompassing single-agent doxorubicin, doxorubicin combined with ifosfamide, and other protocols that varied in dosage, treatment duration, and administration methods. Furthermore, the absence of thorough subgroup analyses, for instance by disease grade or margin status, is a notable limitation likely attributable to the rarity of UPS. This omission could mask a genuine treatment benefit within specific patient subgroups. Finally, while the reported OS benefit is statistically significant, it translates to a modest absolute improvement of only 6%. Moreover, we noticed both the A + I and G + D regimens are associated with limited response rates, and OS remains unsatisfactory (37, 38). Adjuvant radiotherapy is also commonly employed, particularly for high-grade tumors or cases with close or positive surgical margins (25). Therefore, the dosages of chemotherapy and radiotherapy for UPS require careful consideration based on individual patient factors, including specific condition, pathological grade, and margin status. In our case, the elderly patient had a history of coronary heart disease, coronary stent implantation, and HBV infection. A preoperative multidisciplinary collaboration thoroughly reviewed these conditions, and the patient was informed of the surgical risks, potential complications, and the possibility of HBV reactivation following surgical trauma. Such reactivation could lead to fulminant hepatitis, acute liver injury, renal function deterioration, or multiple organ failure. The patient also expressed concern about developing severe gastrointestinal symptoms during radiotherapy and chemotherapy, fearing these effects might be intolerable or life-threatening. As a result, he ultimately declined radiotherapy and chemotherapy.

UPS exhibits multiple genetic mutations and aberrant signaling pathways, highly similar to those of HCC (39, 40), which reveal new opportunities for targeted drug therapies (17, 19, 20). Anlotinib, a novel tyrosine kinase inhibitor, acts on several components of VEGF/VEGFR signaling and fibroblast growth factor receptors; it demonstrates antitumor activity with manageable toxicity in UPS, yielding a median progression-free survival (PFS) of 4.1 months and OS of 11 months following anthracycline-based chemotherapy (41). A multicenter retrospective real-world analysis similarly reported a disease control rate of 59.6% and acceptable efficacy and tolerability for pazopanib in patients with advanced UPS previously treated with cytotoxic chemotherapy (42). In our case, we identified a TP53 mutation, consistent with the findings of Suzuki et al. (43). TP53 functions as a transcription factor that stabilizes under genotoxic stress (44), inducing the transcription of genes involved in cell cycle arrest, apoptosis, and metabolism to exert its tumor-suppressive role. Most TP53 mutations are missense mutations within the DNA-binding domain, causing mutant TP53 to lose its tumor-suppressive activity and acquire oncogenic functions independent of wild-type TP53 (wtp53) (45). Notably, a recent study observed that 18.4% of tumors exhibited gene loss at the TP53 locus, while 32% carried TP53 mutations (46). Moreover, a majority of tumors without TP53 alterations show deletion or silencing of the p14ARF gene, a negative regulator of MDM2 (46, 47). These observations indicate that the p14ARF-MDM2-p53 pathway plays a critical role in UPS pathogenesis. Restoring wtp53 activity may therefore offer a therapeutic strategy for UPS. The TERT gene promotes cancer cell immortalization by serving as the catalytic component of the telomerase complex (48). TERT promoter mutations represent one of the most frequent somatic genetic alterations in human cancers. Although TERT promoter hotspot mutations are uncommon across all sarcomas, they occur relatively often in myxoid liposarcoma (74% of cases) but are rare in UPS (49). Such mutations can reactivate telomerase in sarcomas, potentially conferring unlimited proliferative potential (50). Consequently, interventions targeting telomerase may represent a promising future direction for sarcoma therapy.

The tumor microenvironment significantly influences responses to ICIs. Tumors with mismatch repair deficiency (dMMR) display distinct genetic features and arise most frequently in gastric and colorectal cancers, while occurring less often in other malignancies (51). These tumors accumulate 10 to 100 times more mutations than those with proficient mismatch repair systems (52). This hypermutability particularly affects repetitive DNA sequences, or microsatellites, resulting in high microsatellite instability (MSI-H). Key genes governing the mismatch repair pathway are MLH1, MSH2, MSH6, and PMS2 (53). Cells from dMMR tumors frequently express PD-L1 on their surface. Microscopic examination also reveals substantial lymphocytic infiltration in these tumors, and these immune cells commonly exhibit up-regulated checkpoint proteins such as PD-1,CTLA-4,and lymphocyte activation gene 3 (54). Research has identified PD-L1 expression in UPS that correlates with T cell infiltration. This observation suggests that UPS may align with the ‘inflamed tumor’ paradigm and could explain the efficacy of single-agent PD-1 antibodies (55). Among the samples evaluable for tumor response, PD-L1 expression was detected in only two cases (4%). Both of these tumors were UPS and subsequently responded to treatment. Similarly, the SARC-028 trial found that only three of 80 advanced sarcoma biopsy specimens were PD-L1 positive, and all three were UPS (56). These collective findings imply that UPS patients with PD-L1 expression represent the subgroup most likely to benefit from anti-PD-1 therapy.

Although ICIs remain in the exploratory phase for sarcoma, studies have already yielded promising results. Pembrolizumab is a humanized immunoglobulin G4 (IgG4) monoclonal antibody targeting the PD-1 inhibitory immune checkpoint receptor on lymphocytes. By blocking PD-1’s interaction with its PD-L1 and PD-L2 ligands, the antibody reactivates T cell-mediated tumor destruction (57). It has demonstrated efficacy in advanced colorectal cancer and other MSI-H solid tumors (58). The SARC-028 trial of pembrolizumab in advanced sarcoma reported an overall response rate (ORR) of 17.5% (7/40) across all soft tissue sarcomas (STS) and 40% (4/10) specifically in UPS (56). This indicates a significantly better therapeutic response for UPS compared to other STS subtypes. However, only one of the ten UPS patients achieved complete remission, while three showed partial remission, and disease progression occurred in 30% of cases. Arora et al. (59) described a UPS patient with multiple lung and lymph node metastases following surgery and chemotherapy. After three months of combined pembrolizumab and pazopanib, the lung metastases regressed. Ten months after initiating combination treatment, the patient remained in good overall condition without Grade 3 or 4 severe adverse reactions. Nivolumab, a PD-1 inhibitor, and ipilimumab, which blocks CTLA-4, have also been evaluated in metastatic sarcoma (60). A phase 2 trial found that 37 of 42 evaluable patients (88%) in the nivolumab monotherapy group experienced disease progression. Their median PFS and OS were 1.7 months and 10.7 months, respectively, with a 1-year OS rate of 40.4%. In the combination group (nivolumab plus ipilimumab), 20 of 41 evaluable patients (48.8%) were alive, with median PFS of 4.1 months, median OS of 14.3 months, and a 1-year OS rate of 54.6%. The remission rate with combination therapy was at least comparable to that of chemotherapy regimens based on doxorubicin, gemcitabine, or docetaxel. This efficacy in a large, unselected sarcoma cohort suggests that nivolumab combined with ipilimumab represents a viable second-line treatment. Together, these findings indicate that undifferentiated pleomorphic sarcoma is an immunoreactive STS subtype particularly responsive to ICIs. Future clinical trials incorporating biomarkers such as PD-L1 expression could help identify patients most likely to benefit from immunotherapy.

For this patient, our team encountered considerable diagnostic and therapeutic challenges preoperatively, from which a favorable experience was gained. Initial assessment suggested HCC, given the patient’s age, hepatitis B virus history, and elevated AFP; however, imaging revealed atypical features, including persistent enhancement rather than the classic wash-in and wash-out pattern, along with extensive necrosis and heterogeneous signal intensity. The elevated AFP may have resulted from active hepatitis B virus infection despite undetectable HBV DNA, or from tumor rupture. This case underscores that in HBV-positive elderly males with raised AFP yet non-classical imaging, broader differential diagnose-including rare tumors-should be considered. Spontaneous rupture and hemorrhage necessitated surgical intervention, given the tumor’s aggressive behavior and acute complications. IHC demonstrated PD-L1 expression in the UPS, leading to adjuvant treatment with pembrolizumab according to sarcoma guidelines. The combination of surgery and immunotherapy successfully managed both the hemorrhage and the malignancy, resulting in a favorable clinical outcome. Obviously, as a single case report, the clinical significance and generalizability are inherently limited, and conclusions regarding therapeutic efficacy require further large-sample, multi-center research.

## Future directions

Immunotherapy has shown extremely promising research results in the study of sarcomas. There is a clear need to develop predictive biomarkers to identify sarcomas most likely to benefit from checkpoint blockade. Future phase III clinical trials should focus on specific sarcoma subtypes with baseline tumor-infiltrating lymphocyte infiltration, as well as subtypes that have already shown clinical efficacy with checkpoint inhibitors, such as UPS, angiosarcoma, and myxofibrosarcoma. Selecting these sarcoma subtypes may ultimately improve the efficacy observed in this trial. Incorporating relevant analyses, such as PD-L1 testing and tumor mutation burden assessment, is essential.

## Data Availability

The datasets presented in this study can be found in online repositories. The names of the repository/repositories and accession number(s) can be found in the article/**Supplementary Material**.
