# Supplementary figures and images for: Pembrolizumab combined with surgical treatment for spontaneously ruptured undifferentiated pleomorphic sarcoma of the liver: a case report and literature review
Source: Front Immunol. 2025 Nov 10;16:1691575. doi: 10.3389/fimmu.2025.1691575 (PMC12640869; doi:10.3389/fimmu.2025.1691575)

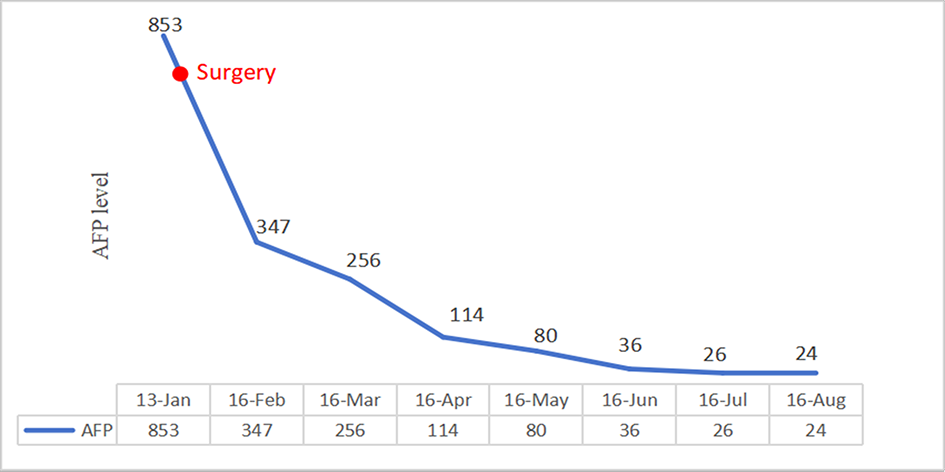

Supplement: Supplementary file 1 [file Image1.tif]
